# Impact of Transgenic Insect-Resistant Maize LD05 on Rhizosphere Soil Bacterial Communities

**DOI:** 10.3390/microorganisms14030718

**Published:** 2026-03-23

**Authors:** Wenlan Li, Xiaohui Xu, Xinwei Hou, Runqing Yue

**Affiliations:** 1 Shandong Key Laboratory of Maize Biological Breeding, Maize Research Institute, National Engineering Center of Wheat and Maize, Shandong Academy of Agricultural Sciences, Jinan 250100, China; liwenlantutu@126.com (W.L.); houxinwei92@163.com (X.H.); 2 Shandong Key Laboratory of Plant Virology, Institute of Plant Protection, Shandong Academy of Agricultural Sciences, Jinan 250100, China; xuxiaohui1023@163.com

**Keywords:** insect-resistant transgenic maize, bacteria, community, rhizosphere soil, diversity

## Abstract

The artificially modified *Bacillus thuringiensis* (Bt) protein can target lepidopteran pests, and planting genetically modified crops with insect-resistant traits is environmentally friendly. However, it is still uncertain whether the exogenous insect-resistant proteins in genetically modified crops will affect the soil rhizosphere microorganisms. This study utilized 16S rDNA sequencing technology to analyze the rhizosphere soil of insect-resistant genetically modified corn LD05 and its control variety Zheng58 at five developmental stages: before sowing, seedling stage, jointing stage, silk emergence stage, and maturity stage. Each sample was taken with six biological replicates, resulting in a total of 60 sequencing samples, with an average of 4368 OTUs obtained per sample. Both alpha and beta analyses showed that LD05 and Zheng58 did not have a significant impact on the soil rhizosphere microbial community. The developmental stage rather than the variety was the main factor causing differences in the bacterial community. Overall, there was no significant difference in the bacterial diversity between the insect-resistant genetically modified corn LD05 and its control variety Zheng58. The results provide useful information for understanding the impact of genetically modified crops on soil microbial communities and also provide a theoretical basis for the safety evaluation of LD05.

## 1. Introduction

Since the United States first commercialized genetically modified crops in 1996, various types of genetically modified crops have developed rapidly, including insect-resistant, herbicide-resistant, virus-resistant, and multi-resistant genetically modified crops [1,2]. The global planting area of genetically modified crops has increased from 1.7 million hectares in 1996 to 202.2 billion hectares in 2022 [3]. Insect-resistant genetically modified crops usually carry more than one insect-resistant gene, which can target and kill lepidopteran pests. Consequently, no additional insecticides need to be sprayed throughout the entire growth period of corn, significantly reducing economic and labor costs [4,5]. This is very beneficial for the sustainable development of society and the environment [4].

Although the cultivation of genetically modified crops has brought significant economic benefits in agricultural production, their potential environmental impacts have increasingly attracted widespread attention from the academic community and society [6]. Among them, the soil microbial community is an important component of the soil ecosystem [7]. The impact of genetically modified crop cultivation on the soil microbial community has become a research hotspot. During the entire growth period of genetically modified crops and after the residue is returned to the soil, the Bt protein in them will enter the soil through secretions, pollen and residues [8,9]. Compared to the entire soil, the content level of Bt protein in the rhizosphere soil and the activity of the microbial community have both increased [10]. Therefore, changes in the structure and diversity of the rhizosphere microbial community are widely recognized as early and effective indicators for evaluating the potential impacts of genetically modified crops on soil ecology [11,12,13].

Previous studies have demonstrated that the Bt protein naturally produced by *Bacillus thuringiensis* itself exerts no adverse effects on the environment [14]. In contrast, the artificially modified Bt protein expressed in transgenic crops has strong toxicity and targets lepidopteran pests, and there are differences in whether it has an impact on the environment. For instance, the cultivation of Bt transgenic corn varieties Bt11 and Mon810 has been shown to significantly change the composition, diversity and richness of the fungal community in the rhizosphere soil [15,16]; similarly, the planting of transgenic rapeseed and cotton harboring the Cry1Ac gene had a significant impact on the diversity, richness and evenness of the rhizosphere microbial populations [17,18]. However, other studies have reported contrasting results, such as the planting of transgenic eggplant containing Cry1Ac, transgenic corn MON863 containing Cry3Bb, and transgenic cotton containing Cry1Ac and CpTI [19,20,21], indicating that planting of most transgenic insect-resistant crops has no significant impact on the soil microbial community. An increasing number of researchers believe that the scientific assessment of whether transgenic crops affect soil ecosystems should fully consider the specificity of each transgenic plant and should be analyzed according to a “case-by-case principle” [22,23].

*m2cryAb-vip3A* is a novel fused insect-resistant gene with independent intellectual property rights [24]. The expressed protein possesses the activities of both Cry1Ab and Vip3A insecticidal proteins [24]. The transformed plant LD05 obtained using this gene has been granted a safety certificate for agricultural genetically modified organisms in China, showing high resistance to *Ostrinia furnacalis* (Guenée), *Mythimna separata* (Walker), *Helicoverpa armigera* (Hübner), and *Spodoptera frugiperda* (J. E. Smith) [24,25]. This study focuses on the effects of the insect-resistant corn LD05 and the control variety Zheng58 on the soil rhizosphere microbial community. The 16s rDNA sequencing technology was used to investigate the abundance, composition, and diversity of the soil rhizosphere microbial community. The results will provide useful information for the biosafety management of the insect-resistant transgenic corn LD05.

## 2. Materials and Methods

### 2.1. Plant and Soil Materials

The insect-resistant transgenic corn LD05 and the non-transgenic control variety Zheng58 were provided by the Shandong Academy of Agricultural Sciences. The experimental site is located in Jinan, Shandong Province, China (N 36°41′50″, E 117°04′33″) in 2024, and the planting adopts a randomized block design. This region features a warm temperate, semi-humid continental monsoon climate, predominantly governed by the East Asian monsoon circulation. Its defining climatic attributes include well-differentiated seasonal transitions, strong coupling between precipitation and thermal regimes (i.e., rainfall and high temperatures co-occurring during summer), high solar irradiance, and pronounced spatiotemporal regularity in key meteorological variables—including temperature, precipitation, and sunshine duration. Each corn variety was set up with three replications. The soil at the experimental site is cinnamon soil, with a weakly alkaline to neutral pH, and the fertilizer application level is at a medium level. Seven days before planting, 3000 kg of organic fertilizer and 30 kg of compound fertilizer containing nitrogen (N), phosphorus (P), and potassium (K) were applied per acre, and these fertilizers were buried 18 cm deep in the soil. No pesticides or herbicides were used throughout the growth period. Soil samples from the rhizosphere of the plants were collected at five different developmental stages: the pre-seeding period, seedling stage, jointing stage, silk emergence stage, and maturity stage [26]. These samples were quickly transferred to liquid nitrogen and then stored at −80 °C.

### 2.2. DNA Extraction and Sequencing of 16S rDNA Amplification

Rhizosphere soil DNA was extracted using the soil genomic DNA extraction kit (DP336, Tiangen, Beijing, China) following the kit instructions, and its concentration was measured with a Nanodrop 2000 (Thermo Fisher Scientific, Wilmington, DE, USA).

The full-length 16S rRNA gene was amplified from the genomic DNA of each sample using universal primers (27F: AGRGTTTGATYNTGGCTCAG and 1492R: TASGGHTACCTTGTTASGACTT) [26], and sample-specific PacBio barcode sequences were added to the tails of both the forward and reverse 16S primers. The PCR amplification mix used was KMM-101, produced by Toyobo, Osaka, Japan. The amplification conditions were as follows: pre-denaturation at 95 °C for 2 min, followed by 25 cycles of denaturation at 98 °C for 10 s, annealing at 55 °C for 30 s, and extension at 72 °C for 2 min. All PCR products were purified using Agencourt AMPure XP Beads (Beckman Coulter, Indianapolis, IN, USA) and quantified using the Qubit dsDNA HS Assay Kit and Qubit 4.0 Spectrophotometer (Thermo Fisher Scientific, Oregon, OR, USA). SMRTbell processed the amplified DNA using the SMRTbell Express Template Preprocessing Kit 2.0 (Pacific Biosciences of California, Inc., Menlo Park, CA, USA) according to the manufacturer’s specifications. Subsequently, the purified SMRTbell libraries were sequenced on a single PacBio Sequel II system.

### 2.3. Bioinformatic Analysis

Most of the bioinformatic analyses were conducted using the BMK cloud platform (a biomarker technology company located in Beijing, China). (1) Sequencing data processing: The raw data were filtered and de-grouped using the SMRT Link software v13.1, with a minimum predicted accuracy set at 0.9 and a minimum pass count set at 5, which served as the standard to obtain circular consensus sequencing (CSS) reads. Through filtering, trimming, and deletion of low-quality reads, unique reads were obtained. (2) OTU clustering: Sequences with a similarity of 97% or more were clustered into one operational classification unit (OTU). The classification annotation of OTUs was assigned based on the naive Bayes classifier in QIIME2 [27] at a confidence threshold of 70%. (3) Sample complexity analysis (alpha diversity): The alpha diversity was calculated and plotted using QIIME2 and R software. (4) Comparative analysis of multiple samples (beta diversity): The UniFrac distance between different samples was calculated based on the OTU table. The beta diversity was analyzed using PCoA analysis with the R packages vegan and ggplot2. (5) Biomarker identification: The analysis was conducted using the linear discriminant analysis effect size (LEfSe) of PERMANOVA. Based on 999 permutations, a network tool (https://bioincloud.tech/standalone-task-ui/lefse, accessed on 16 May 2023) was used to compare between two adjacent growth stages to identify biomarkers (LDA > 3).

### 2.4. Statistical Analysis

All statistical analyses were conducted using the R software (version 4.1.0). The significant differences between the two groups were calculated using Student’s *t*-test based on the *p*-value. For significant differences between more than three groups, the Permutation Multivariate Analysis of Variance (PERMANOVA) was employed. In the linear discriminant analysis effect size (LEfSE) section, the LDA value was set to 3.0 to search for microbial biomarkers.

## 3. Results

### 3.1. Overview of Sequencing of 16S rDNA Amplification

**Figure 1:**
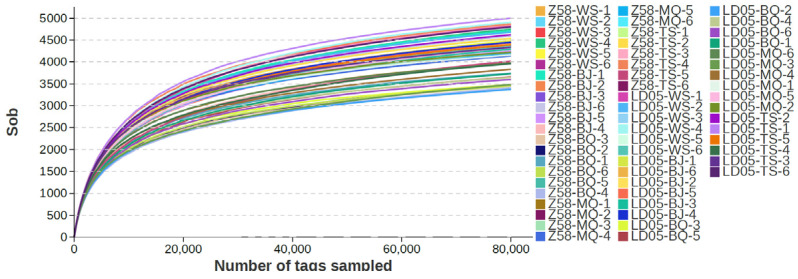
Rarefaction curve of LD05 and Z58 at different periods. BQ: Pre-seeding period; MQ: seedling stage; BJ: jointing stage; TS: silk emergence stage; WS: maturity stage. Z58: Zheng58.

**Figure 2:**
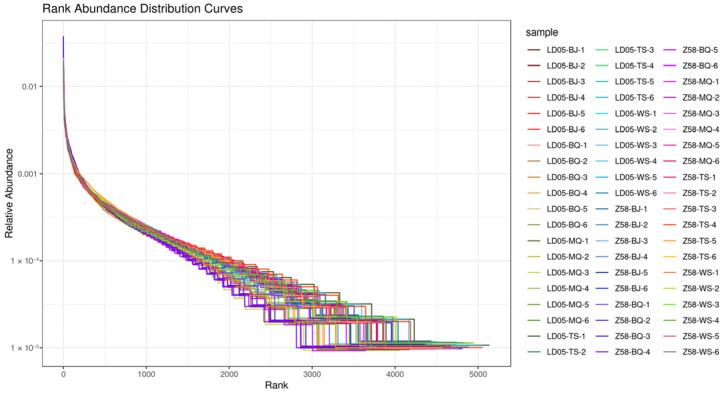
Rank abundance of LD05 and Z58 at different periods. BQ: Pre-seeding period; MQ: seedling stage; BJ: jointing stage; TS: silk emergence stage; WS: maturity stage. Z58: Zheng58.

**Figure 3:**
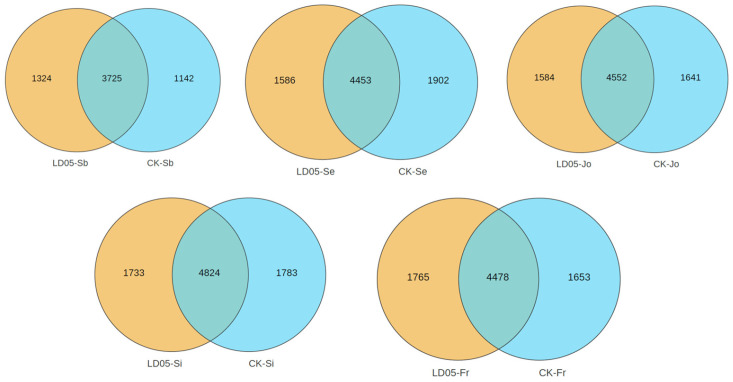
Venn diagrams of LD05 and Z58 at different periods. Sb: Pre-seeding period; Se: seedling stage; Jo: jointing stage; Si: silk emergence stage; Fr: maturity stage. CK: Zheng58.

By conducting 16S rDNA sequencing on the rhizosphere soil of transgenic corn LD05 and non-transgenic control corn Zheng58 during five developmental stages—pre-seeding period, seedling stage, jointing stage, silk emergence stage, and maturity stage—with six biological replicates taken for each sample, a total of 60 sequencing samples were obtained. The results are shown in Table 1. On average, each sample yielded 128,523 raw reads (minimum 120,073, maximum 137,816) and 128,466 high-quality reads (minimum 120,027, maximum 137,753). After performing multiple-sequence alignment on the high-quality sequences and clustering based on a 97% similarity threshold, each sample averaged 4368 OTUs (minimum 3525, maximum 5138). The dilution curve (Figure 1) and abundance curve (Figure 2) showed a gradually flattening trend as the sequencing volume increased, indicating a uniform distribution of species and reasonable data volume, which is suitable for subsequent scientific research analysis. The numbers of shared OTUs between LD05 and Zheng58 were 3725 at the pre-sowing stage, 4453 at the seedling stage, 4552 at the jointing stage, 4824 at the silking stage, and 4478 at the maturity stage, respectively (Figure 3).

### 3.2. Alpha and Beta Diversity of Rhizosphere Bacterial Community of Transgenic Maize LD05 and Its Control

**Figure 4:**
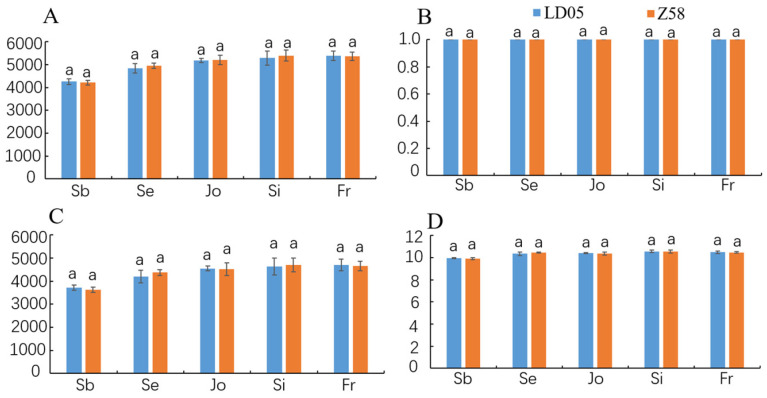
Comparative analysis of four alpha diversity indices in rhizosphere bacterial communities from transgenic maize LD05 and its control. Z58: Zheng58. (**A**) ACE index; (**B**) Simpson index; (**C**) Sobs index; (**D**) Shannon index. Different lowercase letters above error bars indicate a significant difference in different treatments by Duncan’s multiple range test at the 0.05 level.

The alpha diversity indices, including ACE, Simpson, Sobs and Shannon, were used to evaluate the differences in the rhizosphere bacterial communities between the transgenic corn LD05 and its control variety Zheng58. As shown in Figure 4, no significant effect of the transgenic insertion on the bacterial community index was observed at the same developmental stage.

**Figure 5:**
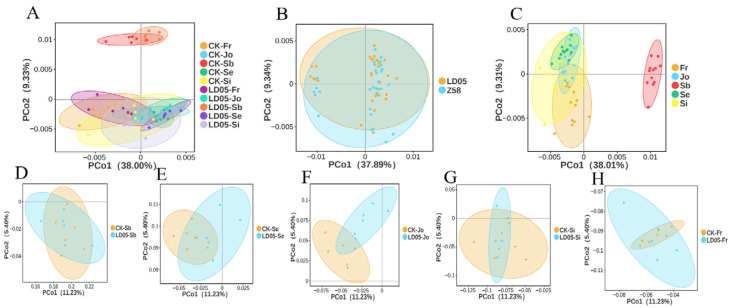
(**A**) PCoA analysis based on UniFrac distances was conducted to characterize the rhizosphere bacterial communities of transgenic maize LD05 and its control at various growth stages. (**A**) PCoA using two variables: different growth stages and maize inbred lines; (**B**) PCoA using one variable: different maize inbred lines; (**C**) PCoA using one variable: different growth stages; (**D**–**H**) PCoA using different maize inbred lines as a variable at pre-seeding period (**D**), seedling stage (**E**), jointing stage (**F**), silk emergence stage (**G**), and maturity stage (**H**). The adonis R value represents the overall variation that can be explained by a certain grouping pattern in the PCoA analysis. Sb: Pre-seeding period; Se: seedling stage; Jo: jointing stage; Si: silk emergence stage; Fr: maturity stage. Z58: Zheng58.

Principal coordinate analysis (PCoA) was performed with two variables, maize growth stages and inbred lines, and the results are shown in Figure 5A. There were significant differences in the bacterial communities in the rhizosphere soil of different maize varieties under different growth stages (*p* = 0.001). To explain the key factors causing these differences, we analyzed the effects of developmental stages and different maize varieties on the diversity of bacterial communities separately. The results indicated that the differences among different maize varieties did not lead to significant changes in the bacterial community (Figure 5B). However, when developmental stage was taken as the sole variable, there were significant differences among the bacterial communities of different developmental stages (*p* = 0.001) (Figure 5C). Specifically, the principal coordinate analysis showed that there were no significant differences in the β-diversity of the bacterial communities in the rhizosphere soil of the two maize varieties in the five growth stages (Figure 5D–H). Therefore, we believe that the main factor influencing the changes in the diversity of rhizosphere bacterial communities is the growth stage, rather than the maize variety.

### 3.3. Bacterial Community Composition

**Figure 6:**
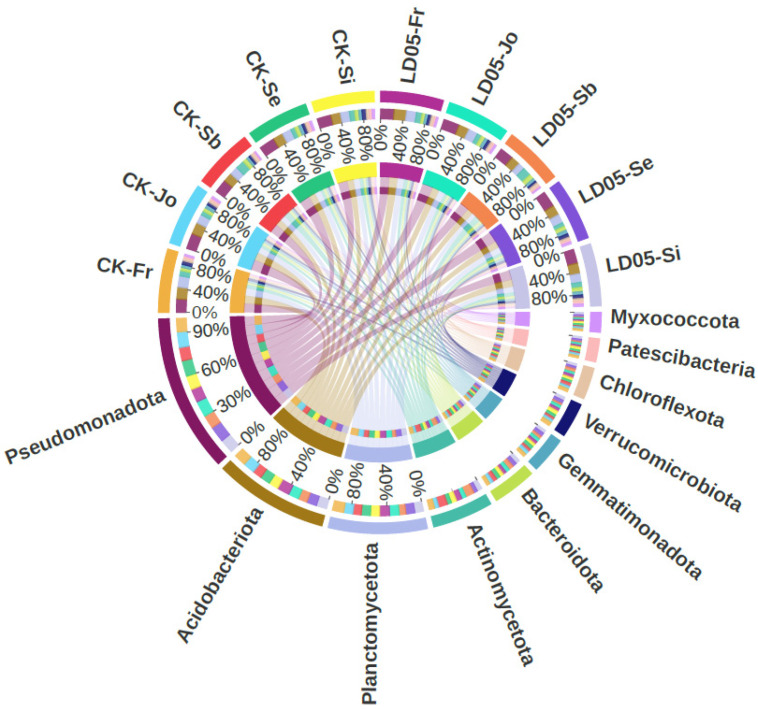
A chord reflecting taxonomic abundance information on the top ten bacterial phyla in the rhizosphere of LD05 and control Zheng58 (CK) at different stages. Sb: Pre-seeding period; Se: seedling stage; Jo: jointing stage; Si: silk emergence stage; Fr: maturity stage.

The bacterial communities were analyzed at different classification levels to determine the types of microorganisms that might exist in the rhizosphere soil zones. The top 10 bacterial phyla that accounted for the largest proportion among all the sequences were: Pseudomonadota, Acidobacteriota, Planctomycetota, Myxococcota, Patescibacteria, Chloroflexota, Verrucomicrobiota, Gemmatimonadota, Bacteroidota and Actinomycetota (Figure 6).

**Figure 7:**
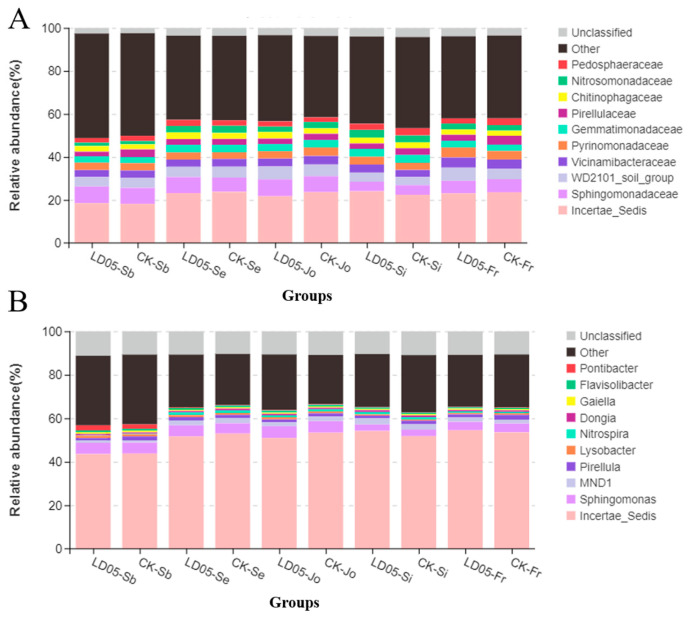
The dominant community structures at the family (**A**) and genus (**B**) levels of root-associated soil microorganisms in transgenic maize LD05 and non-transgenic control variety Zheng58 during different developmental stages. CK in the figure represents the non-transgenic control variety Zheng58. Sb: Pre-seeding period; Se: seedling stage; Jo: jointing stage; Si: silk emergence stage; Fr: maturity stage.

Further analysis of the microbial community structure of transgenic corn LD05 and its control variety Zheng58 revealed that, in the same sampling period, the compositions of the microbial families and genera in the rhizosphere soil of the two corns were basically the same (Figure 7). The dominant bacterial communities at the family level were: Incertae Sedis, Sphingomonadaceae, Chitinophagaceae, Gemmatimonadaceae, WD2101 soil group, vicinamibacteraceae, etc. (Figure 7A). The dominant bacterial communities at the genus level were: Incertae Sedis, Sphingomonas, MND1, Pirellula, Nitrospira, Flavisolibacter, etc. (Figure 7B). The results indicate that there was no significant difference in the bacterial community composition between the transgenic corn LD05 and its control variety Zheng58 at the family or genus level.

**Figure 8:**
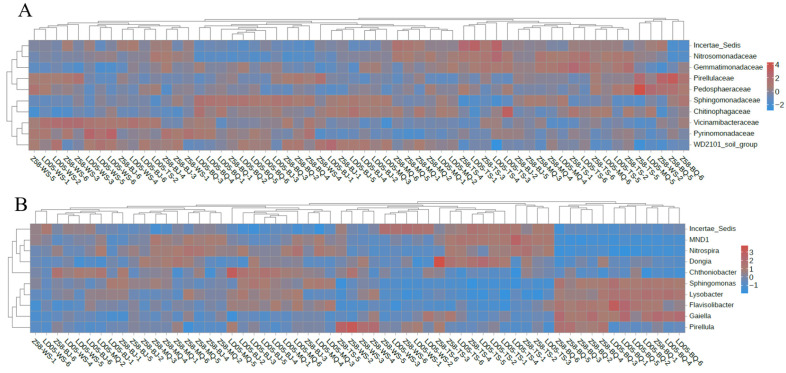
Hierarchical clustering heatmaps displaying the taxonomic abundance of bacterial communities at the family (**A**) and genus (**B**) levels in the rhizosphere of LD05 and Z58 at different stages. BQ: Pre-seeding period; MQ: seedling stage; BJ: jointing stage; TS: silk emergence stage; WS: maturity stage. Z58: Zheng58.

Furthermore, we also drew cluster heatmaps of relative abundance for the first 10 families and the first 10 genera (Figure 8). We compared the species abundance of transgenic corn LD05 and its control Zheng58 at the family level and genus level. The results showed that there was no significant difference between transgenic corn LD05 and its control Zheng58 during the same sampling period.

### 3.4. Identification of Biomarkers Among Bt-Transgenic and Control Maize Lines

**Figure 9:**
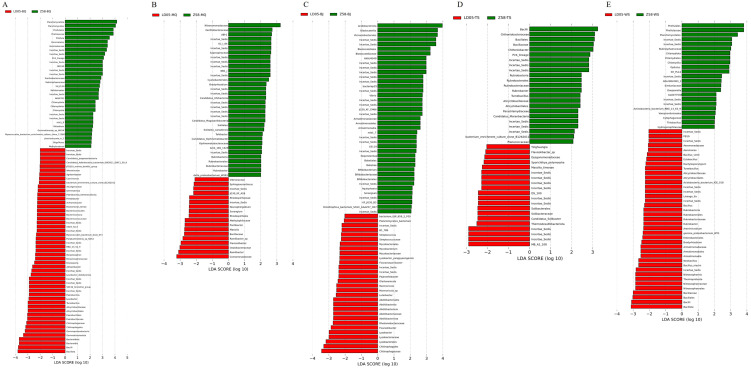
LDA scores of discriminative bacteria in the rhizosphere samples between LD05 and Zheng58 (Z58). (**A**) Pre-seeding stage; (**B**) seedling stage; (**C**) jointing stage; (**D**) silk emergence stage; (**E**) mature stage.

Using the LEfSe analysis method at the phylum level, the bacterial biomarkers that play a role in the changes in root-associated bacteria composition in the different developmental stages of transgenic corn LD05 and its control variety Zheng58 were determined. In the pre-seeding stage, the main bacteria of LD05 were Bacillota, and those of Zheng58 were Planctomycetota; in the seedling stage, the main bacteria of LD05 were Comamonadeceae, and those of Zheng58 were Nitrosomonadaceae; in the jointing stage, the main bacteria of LD05 were Chitinophagaceae, and those of Zheng58 were Acidobacteriota; in the silk emergence stage, the main bacteria of LD05 were MB_A2_108, and those of Zheng58 were Bacilli; in the mature stage, the main bacteria of LD05 were Bacillota, and those of Zheng58 were Pirellulales (Figure 9).

## 4. Discussion

The soil microorganisms in the rhizosphere are mainly composed of bacteria, which can convert inorganic substances into organic substances and promote the growth and development of plants; the roots of plants can also provide nutrients and energy sources for the rhizosphere microorganisms [28,29]. The composition and abundance of the rhizosphere soil microbial community are affected by various factors such as species, genotype and pH value [21,30]. The exogenous insecticidal proteins expressed by transgenic crops can enter the soil through various pathways, such as root exudates, pollen and residues [31,32,33], and the distribution patterns of exogenous proteins in the soil show different characteristics at different growth stages of plants [34,35,36,37]. Currently, several studies have evaluated the risks of different transgenic crops to the soil ecosystem, but the conclusions obtained are different [5,38], and there is no consensus on whether planting transgenic crops will bring environmental safety issues.

Compared with the influence of seasonal changes, the cultivation of Cry1Ac transgenic eggplants only had a slight impact on the soil bacterial community [9]. The cultivation of Cry1Ah transgenic corn HGK60 did not show significant differences in the composition and diversity of the bacterial community compared to the control at the same growth stage, but there were differences between different growth periods [39,40]. The field residues of Cry3Bb transgenic corn MON863 had no effect on the changes in the bacterial community in the soil, but had a slight impact on the fungal community, mainly due to changes in environmental factors, which were not related to the differences in corn varieties [34]. The analysis results of transgenic corn 2A-7 showed that the developmental stage, rather than the variety, was the most important factor determining the diversity of the bacterial community in the rhizosphere soil. These research results all indicate that the changes in the microbial community in the rhizosphere soil are closely related to environmental factors and seasonal changes, but not related to the cultivation of transgenic crops [9,39,40,41]. LD05 is an insect-resistant and herbicide-resistant transformant with a newly approved safety certificate. The insect-resistant gene *m2cryAb-vip3A* is a new fusion gene that has been artificially modified and optimized, which expands the insecticidal spectrum and can be highly resistant to a variety of Lepidoptera pests such as *Ostrinia furnacalis* (Guenée), *Spodoptera frugiperda* (J. E. Smith) and so on [24,25]. There is no related research on whether the LD05 transformant has an effect on the soil microbial community. Based on previous research, most scholars agree with applying the “case-by-case principle” [22,23]. Therefore, this study employed the third-generation high-throughput sequencing technology to investigate the impact of the cultivation of transgenic insect-resistant corn LD05 on the soil bacteria in the rhizosphere. By analyzing the relative abundance of bacteria at different taxonomic levels, the results indicated that during the same growth and development period, there were no significant differences between the bacterial communities in the rhizosphere of LD05 and its control variety Zheng58 at the phylum, class, order, and other taxonomic levels. Furthermore, the analysis of the bacterial community α and β diversity in the rhizosphere soil of transgenic corn LD05 and its control variety showed that the differences between the two corn varieties were not significant within the same growth period (Figure 4 and Figure 5D–H). However, there were significant differences in samples from different developmental stages (Figure 5C). Therefore, the developmental stage rather than the variety (Figure 5B) is the most important factor determining the diversity of the bacterial community in the rhizosphere soil. This finding is consistent with the results of most previous studies.

However, this study was a one-year field trial, and the changes in soil microbial communities may have long-term cumulative effects. Future long-term site-specific experiments are still needed to further explore the long-term impact of continuous planting of transgenic maize LD05 on soil microbial communities, soil enzyme activities, nutrient cycling and other ecological functions [23]. In addition, this study was mainly focused on bacterial communities rather than fungal communities. As a vital component of soil microorganisms, the response patterns of fungal communities to the transgenic maize LD05 remain to be further explored. Combined with techniques such as ITS gene sequencing, a comprehensive assessment of the overall impact of transgenic maize LD05 on the soil ecosystem should be conducted [42].

In summary, there was no significant difference in the composition and diversity of the bacterial community in the rhizosphere soil between transgenic insect-resistant corn LD05 and its control variety Zheng58. The growth and developmental stage of the crop, rather than the variety type, emerges as the key determinant of rhizosphere soil bacterial community diversity. This finding aligns with the conclusions of most existing studies on the soil microecology of genetically modified crops, further providing a scientific basis for the environmental safety assessment of transgenic maize LD05 and furnishing fundamental data for ecological risk management during its industrialization and promotion.

## 5. Conclusions

The 16S rDNA sequencing technique was used to analyze the composition and diversity of the bacterial communities in the rhizosphere soil of transgenic corn LD05 with the *m2cryAb-vip3A* gene and its non-transgenic control variety Zheng58. Through alpha and beta analyses, it was found that the introduction of exogenous insecticidal proteins did not significantly affect the bacterial communities in the rhizosphere soil. The developmental stage, rather than the variety, was the main factor causing the differences in the rhizosphere bacterial communities.

## Figures and Tables

**Table 1:** Overview of 16S sequencing data of rhizosphere soil from transgenic corn LD05 and non-transgenic control at different developmental stages.

Sampling Period	Sample Name	Raw Reads	Clean Reads	Raw Sigs	Clean Sigs	OTUs
pre-seeding	LD05	128,288	128,233	130,493	126,805	3727
	Zheng58	131,434	131,373	130,446	129,660	3635
seedling stage	LD05	131,476	131,422	130,493	130,070	4197
	Zheng58	128,988	128,940	128,061	127,594	4378
jointing stage	LD05	127,140	127,077	126,119	124,901	4547
	Zheng58	129,712	129,657	128,725	128,240	4514
silk emergence stage	LD05	122,982	122,927	122,048	121,586	4636
	Zheng58	128,029	127,971	127,077	126,665	4704
maturity stage	LD05	127,254	127,193	126,250	125,818	4699
	Zheng58	129,929	129,872	128,908	128,445	4650

## Data Availability

The original contributions presented in this study are included in the article. Further inquiries can be directed to the corresponding author.
